# Directional Paging for 5G Communications Based on Partitioned User ID

**DOI:** 10.3390/s18061845

**Published:** 2018-06-05

**Authors:** Mamta Agiwal, Hu Jin

**Affiliations:** Department of Electrical and Electronics Engineering, Hanyang University, Ansan 15588, Korea; mamta@hanyang.ac.kr

**Keywords:** 5G communications, DRX, UE ID, paging, beams, mmWaves

## Abstract

The millimeter-wave (mmWave) spectrum is one of the important propositions of 5G mobile networks due to its ability to accommodate massive traffic demands and an ever-increasing number of wireless devices. The beam-formed directional technique overcomes the propagation and path loss challenges of mmWave high frequencies. Though the directional convergence is expected to unleash new possibilities, it cannot be implemented with conventional power-saving solutions optimized over omnidirectional communications. Paging broadcast, for users in Idle Discontinuous Reception (IDRX) mode for energy saving, is one such necessary function in a wireless communication that needs modification in highly directional beam-based transmissions. Due to the limited spatial coverage of directional beams, the paging transmission takes place over multiple beams, which increases the paging resource overheads of the network substantially. In this article, we present a novel paging mechanism for the directional air interface in mmWave-enabled 5G communications. Numerical analysis of the proposed Partitioned UE ID-based Directional Paging (PIDP) mechanism reduces the paging resource overheads of the network, resulting in a 15% gain in power savings compared to directional paging transmission without the UE ID partition.

## 1. Introduction

Emerging 5G communications is directly attributable to the increasing bandwidth appetite of the wireless industry, majorly driven by the prevalent use of smart devices, advances in realistic Ultra High Definition (UHD) services, the unabated rise of social media and the need for network solutions for connected living, encompassing: smart cities, healthcare, manufacturing, energy, agriculture, transport, tracking and surveillance. While escalating demands for higher data rates and the popularity of the Internet of Things (IoT) are spawning apparent requirements for high network capacity, at the same time, energy-efficient design is gaining momentum as the fundamental requirement of future wireless networks. However, the emerging millimeter wave (mmWave)-driven complex spectrum techniques raise new challenges for the energy-efficient design aspirations.

The spectral efficiency of microwave links, responsible for all current wireless communications, is approaching its fundamental limit [1], resulting in the progressively growing research interest towards the mmWave radio spectrum for 5G wireless communications. Large chunks of the radio spectrum at mmWave frequencies promise to bridge the gap between the limited “sub-GHz” bandwidth and the desire for high data services and massive connectivity [2]. Although very attractive, the integration of the mmWave spectrum in wireless scenarios is complex, especially in view of high path and propagation losses at higher frequencies. Providentially, small mm wavelengths facilitate embedded antenna array architectures in portable devices. The practical compositions of antenna arrays are capable of focusing electromagnetic energy in desired directions (beams). Beams provide a viable solution to path loss impediments at higher frequencies [3]. Thus, beam-based directional communication is an essential proposition for mmWave-based 5G communications [1,4]. The Third Generation Partnership Project (3GPP) has already started work on efficient alignment of New Radio access technology (NR) designed to operate in the high frequency spectrum (up to 100 GHz) [5].

**Figure 1:**
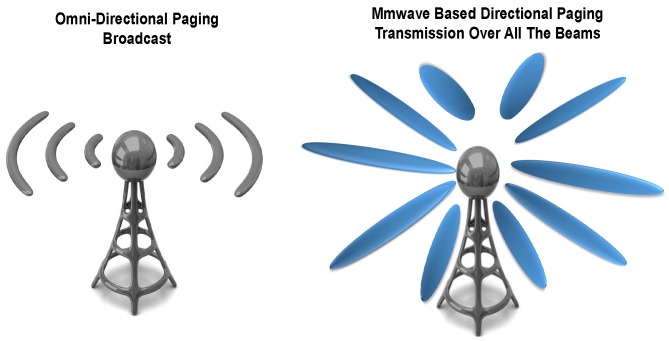
Directional air interface in 5G communications.

Establishing a wireless network using directional beam-based communication is relatively new and different from current omnidirectional wireless communication, as depicted in Figure 1. Directivity changes the way the air interface is processed today, thereby challenging legacy mechanisms designed and optimized for omnidirectional communication. The broadcast transmission of paging messages to User Equipment (UE) in Discontinuous Reception (DRX) mode is one such mechanism that requires redesigning in directional 5G communication. In the LTE/LTE-A wireless system, the DRX mechanism is specified, which reduces the UE’s energy expenditure by configuring sleep periods. During the sleep period, most of the UE’s circuitry is turned off to save battery power [6]. With rising computational expenses at the UE, due to higher order modulation and coding, beam searching overheads, massive MIMO technologies, raised quality expectations and increasing social/multimedia trends, it would be difficult to ignore DRX for energy conservation at the UE in 5G networks [7]. Recently, 3GPP also acknowledged supporting the same in NR [8].

Paging is an important mechanism that helps with directing an incoming call to a UE when it is an Idle mode DRX (IDRX) to conserve energy. Network broadcasts the paging messages in the cells to convey an intimation about the incoming call to the UE in IDRX mode [9]. Since the spatial coverage of each Transmission (TX) beam is limited, the paging message broadcast becomes complicated in directional 5G communications. To cover the area of an entire cell, the paging message needs to be transmitted over all TX beams, as shown in Figure 1. However, simultaneous transmission over all the beams is nonviable due to limited number of antenna panels at the gNB (5G base station is known as next generation NodeB (gNB)) [8]. In mmWave 5G communications, beam sweeping is performed to cover a spatial area by transmitting various beams in different time slots in a deliberate fashion [8,10]. Thus, directional paging transmission over several beams would require multiple time slots to cover the entire cell, while legacy omnidirectional broadcast is possible in only one time slot per Paging Occasion (PO) [10]. This is a crucial challenge since the network’s resource requirement for paging transmission would increase with the increase of the number of beams in directional 5G communications. We present a novel Partitioned UE ID-based Directional Paging (PIDP) mechanism to reduce paging resource overheads in 5G communications. The main contributions of this paper are summarized as follows:

1. The same paging message that is transmitted over several beams is majorly comprised of UE IDs of all the UE, configured in the PO. In PIDP, we propose to reduce the number of UE ID bits included in the paging message to reduce the overall paging resource overheads of the network.
2. We present a novel method for the configuration of POs in the IDRX cycle and an effective distribution of UE over various POs. The proposed configuration of POs in PIDP ensures that the network is able to page all the UE uniquely despite the fact that only partial UE IDs are transmitted in the paging message.
3. We perform numerical analysis to ascertain the advantages of PIDP. Paging resource overheads, the number of bits and time slots required for paging transmission and, subsequently, the base station’s power saving gain are analyzed with respect to variations in the number of beams and paging rate. Recent 5G parameters are considered for the analysis. We compare our proposed PIDP mechanism to directional paging transmission with full UE ID. The proposed PIDP mechanism manifests more than a 15% power saving gain compared to directional paging transmission while using the full UE ID.

By reducing the number of bits included in the paging message transmitted by the network and by efficient reconfiguration of the POs, the PIDP mechanism reduces the network’s paging overheads. It is to be noted that each UE wakes up for a specific PO and monitors only one PO per IDRX cycle. Thus, the PIDP mechanism does not have any negative impact on the UE energy consumption, while at the same time, it achieves power saving in the network. The rest of this paper is organized as follows. In Section 2, we present the related work with a focus on the fundamentals of paging from both the UE’s and network’s perspective. The PIDP mechanism for directional paging and its system model are presented in Section 3. In Section 4, we evaluate the system performance of our PIDP proposal by varying the values of different parameters like UE ID size, number of beams and number of UE per PO. Finally, we draw conclusions in Section 5.

## 2. Related Work

DRX for power saving can be configured in both the Radio Resource Control (RRC) states for a UE: (1) RRC-connected state and (2) RRC-idle state. In an RRC-connected state, the UE remains connected with evolved Node B (eNB). However, DRX configurations navigate the UE to the RRC-idle state, if there is no data activity for long. The network releases the RRC connections and facilitates the UE to enjoy extended sleep. In the RRC-idle state, the UE is free to roam among a group of eNBs in a specific area (i.e., Tracking Area (TA)) such that the handover procedures are prevented. The paging procedure helps in determining the exact cell in which the idle mode UE is located such that the incoming call is redirected properly [11]. The network broadcasts the paging messages in the cells of a TA, to convey the intimation about the incoming call to the UE in IDRX mode [9]. Since paging messages are monitored by the UE and broadcasted by the network, in this section, we highlight the paging complications from both the UE’s and network’s point of view.

### 2.1. Paging Monitoring by the UE

**Figure 2:**
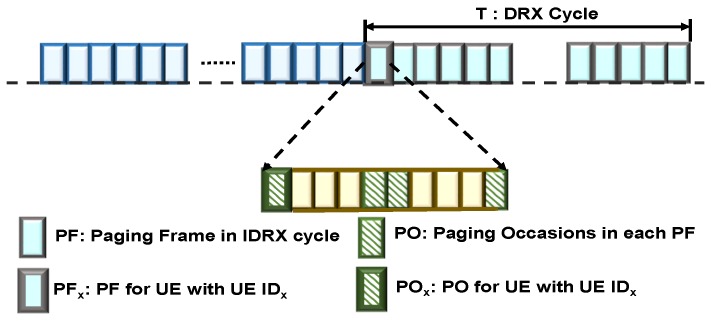
Paging frame (PF) and paging occasion (PO) in a legacy network.

In each IDRX cycle, the UE gets to stay in sleep mode (‘OFF’ duration) to conserve energy. However, the UE is expected to wake up for a specific sub-frame called the Paging Occasion (PO) to monitor the Physical Downlink common Control Channel (PDCCH) for paging. If the PDCCH for paging is received in the PO, then the UE decodes the Physical Downlink Shared Channel (PDSCH) to receive the paging message. If the page is not intended for the UE, it sleeps again till the next PO. Every IDRX cycle, the UE monitors only one PO in a specified Paging Frame (PF). A PF is one radio frame that includes one or multiple POs (up to four in LTE). Figure 2 shows the PFs and POs that can be monitored by the UE with identifier UE ID${}_{x}$. The UE determines the exact timing when it should wake up to receive the paging message that is being sent to it by utilizing PF and PO information [12]. In LTE/LTE(A), the PO and PF that the UE is expected to monitor are elaborated in 3GPP specifications [12]. PF is the radio frame that satisfies:

(1) $$\begin{array}{cc} SFN\;\mathrm{mod}\;T & =(T/N)\times (UEID\;\mathrm{mod}\;N) \end{array}$$

(2) $$\begin{array}{cc} N & =min(T,nB) \end{array}$$

where *SFN* is the System Frame Number and *T* is the DRX cycle of the UE. The term *‘nB’* gives the total number of POs in one DRX cycle and can be any one of T/32, T/16, T/8, T/4, T/2, T, 2T or 4T, the knowledge of which comes from the system information.

Subsequently, the PO is determined as:

(3) $$\begin{array}{cc} i_{s} & =\left(UEID/N\right)\;\mathrm{mod}\;N_{s} \end{array}$$

(4) $$\begin{array}{cc} N_{s} & =max(1,nB/T) \end{array}$$

where $N_{s}$ is the number of POs in a PF and $i_{s}$ indicates the sub=frame number (i.e., PO) in the PF, the value of which is pre-defined for each value of $N_{s}$.

In the proposed PIDP mechanism, we propose to redesign the aforesaid procedure to accommodate a shorter UE ID size in the paging message. A shorter UE ID size would result in the reduction of paging resources in directional 5G communications.

### 2.2. Paging Broadcast by the Network

When in idle mode, the UE performs TA update procedures to notify its location in the network database to the Mobility Management Entity (MME). The paging mechanism is instigated at the MME of the tracking area, and subsequently, the paging message is notified to all eNBs in the TA. Hence, the paging messages are broadcasted in the configured POs for all the cells associated with the last updated TA [10]. Paging procedures along with TA update incur more than 33% of the total signaling load that is managed by the MME in the legacy LTE networks [11]. With the expected increase in the number of mobile subscribers in the near future, the efficient operation of the MME becomes even more crucial. The mmWave beam-formed communication (fundamental to the 5G system) would further accentuate network overheads due to complications of paging broadcast in the directional paradigm. Though several research works with a focus on paging overhead optimization are available, they are based on LTE or MTCcommunications [11,13,14,15,16,17,18,19,20,21]. In [11], paging areas are assigned using mobility models to reduce signaling traffic in LTE. Rule-based artificial intelligence is proposed in [13], while multilevel graph partitioning is promoted in [14] for intelligent paging and TA optimization. In view of the dense and nested deployment of small cells, a local anchor-based location management scheme for HetNets is presented in [15]. For efficient MTC/IoT DRX, novel methods like quick sleeping, group paging and re-synchronization are being researched [18,19,20]. DRX in connected state for 5G is discussed in [7,22,23], and the tracking area management framework for 5G networks is presented in [24]. However, the effect of limited paging resources in the directional air interface is not yet comprehensively studied.

In a directional 5G system, the same paging message needs to be transmitted over all the beams for each cell in the TA. However, the limitation and complexity of antenna modules at the gNB are deterrents to the simultaneous transmission [8] in the cell. The paging transmission takes place over different time slots, resulting in increased resource requirement. The related work in [25] has already shown the overwhelming rise in resources for directional paging in 5G communications in comparison to the legacy LTE networks. Table 1 highlights that the system capacity requirement is substantially high for directional paging in 5G compared to LTE at the 80-MHz bandwidth [25]. In [25], the UE informs about its beam to the gNB by uploading an indication for reducing paging resources. An extension of this work is presented in [10], where the effect of the proposed indication on the energy savings of the UE is evaluated. In [10], the resource savings at the network is achieved at the cost of the increase in energy expenditure at the UE. The increase in energy expense at the UE motivated us to propose the PIDP mechanism, an alternate paging scheme for directional 5G communications. Table 2 summarizes a brief survey of the latest research works in the related fields.

## 3. Proposed PIDP Paging

**Figure 3:**
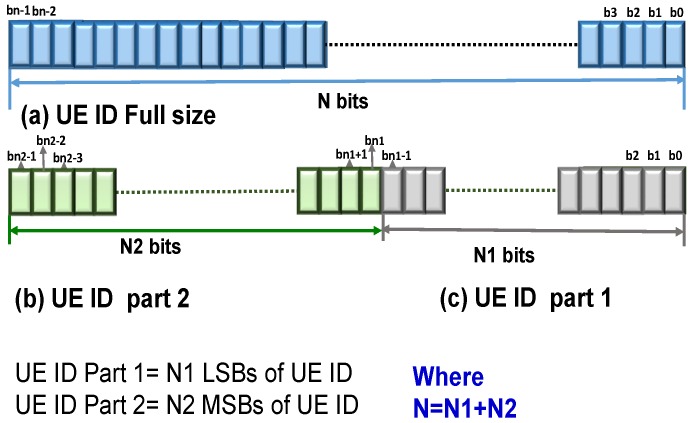
Partitioning of UE ID in the partitioned UE ID-based directional paging (PIDP) mechanism.

In 5G directional communications, due to the limited spatial coverage of each beam, the same paging message is to be transmitted over different beams, resulting in very high paging overhead [25]. In PIDP, we propose to reduce this rising paging overhead in 5G networks by reducing the number of bits included in the paging message being transmitted over different beams. The paging message in every PO is comprised of the UE IDs of all the UE paged in the PO along with RRC overhead bits (e.g., the number of UE IDs and padding), PDCCH bits and Cyclic Redundancy Check (CRC) bits. Network resources required for the broadcast of this paging message further depend on the number of UE and the number of beams. While the number of beams is related to the frequency of operation and the number of UE depends on the user density, the overhead bits are small. Thus, its is inconceivable to reduce beams, overhead bits or user density. Therefore, in this article, we propose to reduce bits for each UE ID included in the paging message, transmitted in every PO, to reduce directional paging overheads. For this, the UE ID is partitioned into two parts as shown in Figure 3. The UE ID is considered to be comprised of *N* bits. Instead of *N* bits, only ‘*N*2’ bits per UE are transmitted in the paging message for all the UE paged in the PO, such that N2<N. The procedures related to the configuration of POs and the distribution of UE over different POs are redesigned in the PIDP mechanism such that the conflicts due to the short UE ID size transmission are avoided. It is thus important to carefully partition the UE ID considering both the bits that would be included in the paging message and also the bits that would be used to configure the PO. Three major steps of the proposed PIDP mechanism, (i) partitioning, (ii) configuring and (iii) distribution, can be elaborated as:

1. Partitioning: The first step of the PIDP mechanism involves partitioning of Nbits of the UE ID into two parts. UE ID Part 1 is comprised of the N1 least significant bits of the UE ID. UE ID Part 2 is made up of the N2 most significant bits of the UE ID. N1 and N2 together provide the complete information of the UE ID as shown in Figure 3.
  (5) $$\begin{array}{cc} N & =N1+N2 \end{array}$$
  We propose that only UE ID Part 2 (comprised of N2 bits) is transmitted in the paging message instead of the entire N bits. Since several UE are configured in each PO and there are multiple POs per IDRX cycle, the reduction in bits to be transmitted per UE would result in the saving of network resources. Moreover, the savings is expected to be substantial as the same paging message is transmitted over several beams.
2. Configuring: It is not always possible to page all UE in the same PO, and it depends on factors like paging load, tracking area size and user density. Thus, multiple POs are required in each IDRX cycle. Since the network is aware of paging load and other factors, it configures numerous POs in the LTE/LTE (A) system. In directional paging, as well, multiple POs would be required for the same reasons. However, in the PIDP mechanism, we propose that the network configures multiple POs in each IDRX cycle based on N1 bits (that is, UE ID Part 1). Let $N_{PIDP}$ be the number of POs that can be configured per IDRX cycle in PIDP, and it is evaluated as:
  (6) $$\begin{array}{cc} N_{PIDP} & =2^{N1}=2^{\left(N-N2\right)} \end{array}$$
  where N1 are the number of bits by which each UE ID size is cropped. The POs are subsequently mapped to all the possible bit configurations that can be attained using N1 bits. Thus, distinct PO in the IDRX cycle has a distinct N1 bit configuration associated with it. Unlike PO/PF evaluation in LTE (Section 2.2), in the PIDP mechanism, we propose a novel and simplified mapping between POs and the least significant bits of the UE ID.
3. Distributing: In the legacy system, multiple POs are configured by the network in the IDRX cycle, and the UE are distributed over these POs. Similarly, in the PIDP mechanism, the UE are distributed over $N_{PIDP}$ number of POs configured for each IDRX cycle. We propose that the UE with the same least significant bits are configured in the same PO. Thus, in PIDP, while only N2 most significant bits of the UE ID (Part 2) are included in the paging message, N1 least significant bits (Part 1) are used to distribute the UE over different POs. This ensures that the IDs of all the UE that are paged are uniquely identified by the network even though only partial bits are included in the paging message.

**Figure 4:**
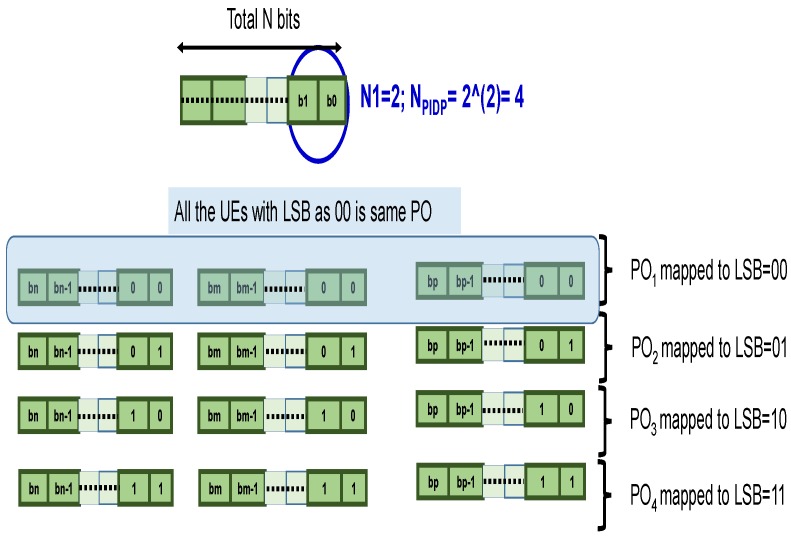
Configuration of paging occasions (POs) and the distribution of UE over POs in PIDP for N1 = 2.

Figure 4 gives an example for the configuration of POs and the distribution of UE over the configured POs when N1=2. For N1=2, four POs are configured each with distinct bit sequence 00,01,10 and 11. All the UE with the same Least Significant Bits (LSBs) (e.g., 00) are distributed over the same PO (e.g., $PO_{1}$). In this example, only N2=N−2 bits per UE are included in the paging message. Though the reduction in the UE ID size per UE is expected to reduce the overall paging resource overheads in directional communication, at the same time, the configuration of POs and the distribution of the UE would also change. It is therefore important to analyze the performance of the PIDP mechanism considering all the related parameters to ascertain its gains. In the subsequent numerical analysis, we evaluate the PIDP gain taking into account the aforesaid partition and configuration.

### PIDP System Model

For PIDP analysis, we first consider the number of POs configured by the network (evaluated using Equation (6). Subsequently, We can evaluate the UE per PO ($U_{PIDP}$) based on the paging rate. Let $R_{pag}$ be the paging rate, defined as the number of UE to be paged per second. The paging rate can be established by accounting for various factors, for instance paging arrival rate, number of gNBs in a tracking area, user density per cell, type of paging scheme, etc. [10]. $U_{PIDP}$ can be evaluated as:

(7) $$\begin{array}{cc} U_{PIDP} & =\frac{R_{pag}\times L_{DRX}}{N_{PIDP}} \end{array}$$

where $L_{DRX}$ is the length of the IDRX cycle in seconds.

The paging message in every PO is comprised of the UE ID of all the UE configured in the PO along with RRC overhead bits ($\epsilon_{RRC}$), CRC bits ($\epsilon_{CRC}$) and PDCCH ($\epsilon_{PC}$) bits. In PIDP, only the partial UE ID (N2: UE ID Part 2 in Figure 3) is included per UE in every PO. Moreover, in the directional interface, the same paging message needs to be transmitted over multiple beams. If $B_{n}$ is the number of beams, then the bits to be transmitted per PO ($b_{PIDP}$) are computed as:

(8) $$\begin{array}{cc} b_{PIDP} & =B_{n}\times \left(U_{PIDP}\times N2+\epsilon_{CRC}+\epsilon_{RRC}+\epsilon_{PC}\right). \end{array}$$

We can calculate the bits that need to be transmitted per second ($b_{s}$) in PIDP as:

$$\begin{array}{c} b_{s}=\mathrm{Bits}\mathrm{to}\mathrm{be}\mathrm{transmitted}\mathrm{per}\mathrm{PO}\times \mathrm{POs}\mathrm{per}\mathrm{IDRX}\mathrm{cycle} \end{array}$$

(9) $$\begin{array}{cc} b_{s} & =b_{PIDP}\times \frac{N_{PIDP}}{L_{DRX}}\\ & =B_{n}\times \left(U_{PIDP}\times N2+\epsilon_{CRC}+\epsilon_{RRC}+\epsilon_{PC}\right)\times \frac{2^{N1}}{L_{DRX}} \end{array}$$

If $\eta_{s}$ is the downlink cell edge spectral efficiency in bits/s/Hz and $\Omega_{w}$ is the system bandwidth in Hz, then the maximum number of bits, $b_{max}$, that can be transmitted in every second is calculated as:

(10) $$\begin{array}{cc} b_{max} & =\eta_{s}\times \Omega_{w}. \end{array}$$

Subsequently, we can evaluate the percentage of system capacity used in the PIDP mechanism ($C_{PIDP}$) when the partial UE ID is used for directional paging in 5G communications as:

$$C_{PIDP}=\left(\frac{\mathrm{Bits}\mathrm{required}\mathrm{to}\mathrm{be}\mathrm{transmitted}\mathrm{per}\mathrm{PO}\mathrm{in}\mathrm{PIDP}}{\mathrm{Maximum}\mathrm{number}\mathrm{of}\mathrm{bits}\mathrm{that}\mathrm{can}\mathrm{be}\mathrm{transmitted}\mathrm{per}\mathrm{PO}}\right)\times 100$$

(11) $$\begin{array}{cc} C_{PIDP} & =\frac{b_{s}\times 100}{b_{max}}\\ & =\left(100\times B_{n}\times \left(U_{PIDP}\times N2+\epsilon_{CRC}+\epsilon_{RRC}+\epsilon_{PC}\right)\times \frac{2^{N1}}{L_{DRX}}\right)/\left(\eta_{s}\times \Omega_{w}\right). \end{array}$$

To ascertain the gains due to PIDP, we also evaluate the percentage of system capacity required when the full UE ID is transmitted for each UE in every PO for directional paging. Moreover, when the full UE ID (comprised of N bits) is transmitted, the network needs to configure only a limited number of POs depending on the paging rate and the maximum possible paging capacity [10]. Let $PO_{N}$ be the minimum number of POs per second required for directional paging that includes the full user ID. We can evaluate $PO_{N}$ as:

(12) $$\begin{array}{cc} PO_{N} & =\frac{N\times R_{pag}}{Cap_{Max}\times L_{DRX}} \end{array}$$

where $Cap_{Max}$ is the maximum paging capacity defined as bits available for paging per time slot of duration *T* seconds. The value of $Cap_{Max}$ is based on the number of bits that is available for transmitting the IDs of paged UE in a time slot for a given carrier with a certain carrier bandwidth. $Cap_{Max}$ can be evaluated as:

(13) $$\begin{array}{cc} Cap_{Max} & =\left(T\times \eta_{s}\times \Omega_{w}\right)-\left(\epsilon_{CRC}+\epsilon_{RRC}+\epsilon_{PC}\right). \end{array}$$

Similar to Equation (9), we can evaluate bits to be transmitted per second $b_{sDP}$ for directional paging with the full user ID for $PO_{N}$ number of POs as:

(14) $$\begin{array}{cc} b_{sDP} & =B_{n}\times \left(\frac{R_{pag}}{PO_{N}}\times N+\epsilon_{CRC}+\epsilon_{RRC}+\epsilon_{PC}\right)\times PO_{N}. \end{array}$$

Subsequently, we can evaluate the percentage of system capacity used in directional paging when the full UE ID is included in the paging message as:

(15) $$\begin{array}{cc} C_{DP} & =\frac{b_{sDP}\times 100}{b_{max}}\\ & =\left(100\times B_{n}\times \left(\frac{R_{pag}}{PO_{N}}\times N+\epsilon_{CRC}+\epsilon_{RRC}+\epsilon_{PC}\right)\times PO_{N}\right)/\left(\Omega_{w}\times \eta_{s}\right). \end{array}$$

We also evaluate the effect of the PIDP proposal on the power savings at the base station. Fewer time slots for paging would facilitate the base station to sleep, resulting in power saving. Let $\psi_{PIDP}$ and $\psi_{DP}$ denote the number of time slots required per second for paging transmission in the PIDP mechanism and directional paging with the full UE ID, respectively. $\psi_{PIDP}$ can be evaluated as:

(16) $$\begin{array}{cccc} \psi_{PIDP} & =(\frac{N_{PIDP}}{L_{DRX}}\times B_{n})/number\;of\;POs\;per\;time\;slot & =\frac{N_{PIDP}\times b_{PIDP}\times B_{n}}{B_{n}\times L_{DRX}\times Cap_{Max}} & =\frac{b_{s}}{Cap_{Max}}. \end{array}$$

Similarly, we obtain $\psi_{DP}=\frac{b_{sDP}}{Cap_{Max}}$. Subsequently, we can evaluate power saving gain ($\Psi_{gain}$) as:

(17) $$\begin{array}{cc} \Psi_{gain} & =\left(\psi_{DP}-\psi_{PIDP}\right)/\psi_{DP}=1-\frac{b_{s}}{b_{sDP}}\\ & =1-\frac{R_{pag}\times N2+\left(\epsilon_{CRC}+\epsilon_{RRC}+\epsilon_{PC}\right)\times 2^{N_{1}}}{R_{pag}\times N+\left(\epsilon_{CRC}+\epsilon_{RRC}+\epsilon_{PC}\right)\times PO_{N}}. \end{array}$$

While $b_{s}$ corresponds to bits required for directional paging with a shorter UE ID, $b_{sDP}$ is evaluated for the full UE ID. Thus, the value of $b_{s}$ is less than $b_{sDP}$. The second term in Equation (17) becomes less than one, and positive power saving gains can be expected for the proposed PIDP mechanism.

## 4. Performance Analysis and Results

The numerical analysis of PIDP is conducted using MATLAB while considering the combined effect of: the reduction in the UE ID size (with respect to N2) and re-configurations of POs (with respect to N1 and the paging rate). In directional communication, the paging resource overhead increases with the number of TX beams since the transmission of the paging message takes place over all the beams. Hence, in this section, we present the performance analysis of PIDP with respect to variations in the UE ID size, paging rate and the effect of the number of TX beams. Various parameters utilized for the analysis of the proposed PIDP mechanism are given in Table 3. In the PIDP performance analysis, the downlink cell edge spectral efficiency is considered as paging is a broadcast communication and is intended to be received by all UE (near the cell edge, as well as at the cell center).

**Figure 5:**
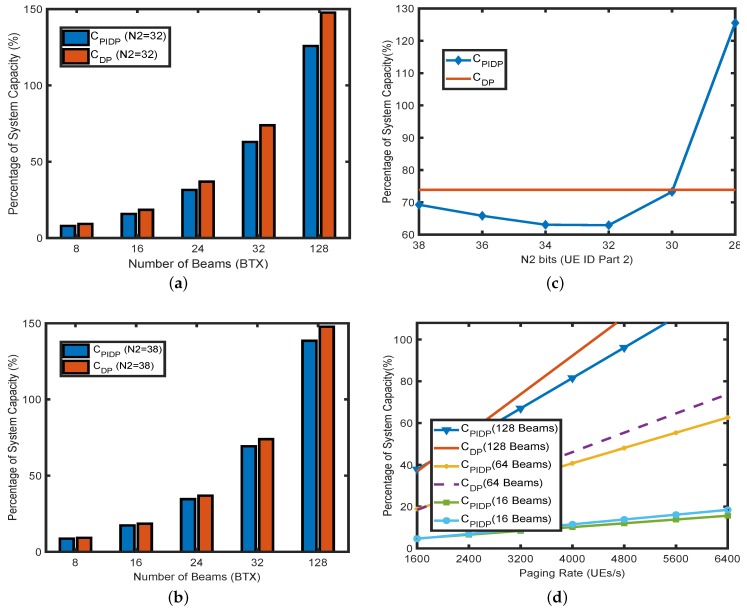
(**a**) Percentage capacity vs. the number of beams (32 bits); (**b**) percentage capacity vs. the number of beams (38 bits); (**c**) Percentage capacity vs. the cropped UE ID size; (**d**) Percentage capacity vs. the paging rate.

We first present the advantage of the PIDP mechanism with respect to variations in number of beams (16, 32, 64 and 128 beams). Figure 5a,b delineates the percentage of system capacity required when the UE ID size reduces by 20% (N2=32) and by 5% (N2=38), respectively, for the PIDP mechanism. Figure 5a,b also shows the comparison of the PIDP mechanism with the percentage of paging capacity required in directional paging with transmission incorporating the full UE ID (N=40) for the paging rate of 6400 UE/s. As the number of beams increases, the paging resource requirement increases since the paging needs to be transmitted over multiple beams (in turn, multiple time slots). However, the increase in $C_{PIDP}$ is less compared to directional paging with the full UE ID, $C_{DP}$. For instance, while directional paging requires almost 73.87% of the system capacity, the requirement reduces to only around 62.9% of system capacity in PIDP for N2=32 and 64 TX beams. As expected, by reducing the number of bits, the paging resource requirement decreases despite the reconfigurations of the number of POs. The reduction in system capacity with N2=32, shown in Figure 5a, is more prominent compared to N2=38 in Figure 5b. The effect of the decrease in N2 is consolidated in Figure 5c.

Figure 5c demonstrates the effect of the decrease in the number of bits of the UE ID per UE (N2) that are included in the paging message for the paging rates of 6400 UE/s when the transmission is executed over 64 TX beams. As the number of bits reduces, the percentage of system capacity initially reduces. However, it shows an overwhelming increase as the bits keep on reducing beyond a threshold (N2=30) in the case of 64 TX beams and 6400 UE/s PR. This is because the network has to configure more POs (Equation (5)) as N2 decreases (N1 increases) to ensure that it is able to uniquely page a UE even though a lesser number of bits is transmitted in the paging message.

The impact of variations in paging rate (UE/s) on the percentage of the system capacity required for paging transmission is highlighted in Figure 5d for both the PIDP mechanism and directional paging with full UE ID transmission. In Figure 5d, the paging rate variations are shown for 16, 64 and 128 number of beams. Moreover, to understand the impact of paging rate on the number of beams, for all the cases, the value of N2 is fixed at 32 in Figure 5d. As the paging rate increases, both $C_{PIDP}$ and $C_{DP}$ increase. For a higher paging rate, the network configures more POs in the case of direction paging with the full UE ID (Equation (12)). At the same time, in the PIDP mechanism, the UE per PO increases with the increase in paging rate (Equation (7)). As a result, a higher percentage of system capacity is required for both $C_{PIDP}$ and $C_{DP}$ with the increase in the paging rate. At a paging rate of 4000 UE/s and for 128 beams, the percentage of system capacity required for paging with the full UE ID is 92.25 in comparison to only 81.56 in the PIDP mechanism.

**Figure 6:**
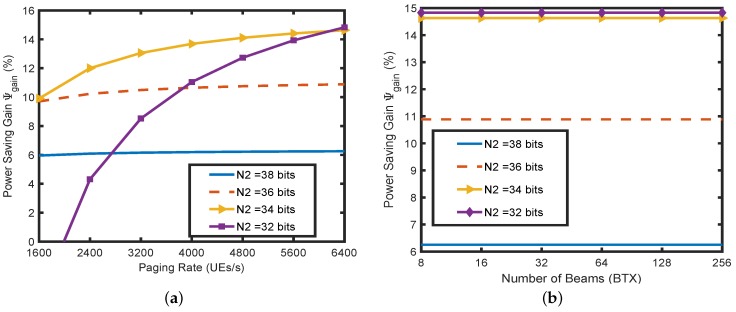
(**a**) Power saving gain vs. paging rate; (**b**) power saving gain vs. the number of beams.

Figure 6a demonstrates the base station’s power saving gain ($\Psi_{gain}$) as a percentage for PIDP with respect to variations in the paging rate for 64 beams. The gain is obtained in comparison to directional paging with the full user ID. Cropped UE ID sizes with values of 32,34,36 and 38 are considered since they are less than the threshold obtained in Figure 5c. As the paging rate increases, the percentage of gain of PIDP increases. It can be observed from Equation (17) that the power saving gain scales exponentially with the factor N1=N−N2. Therefore, while the increase is steeply exponential for lower values of N2 (32), it shows less variations for higher values of N2 (38) when the paging rate increases. For higher N2 (38), the factor $2^{N1}$ becomes less prominent compared to other terms and does not show a drastic effect on the gain percentage. For N2=32 bits, the number of POs configured in PIDP is high ($2^{8}$). When the paging rate is low (1600), the number of POs configured by the network is less in directional paging with the full UE ID. Thus the gain for 32 bits at a lower paging rate is not remarkable. However, as the paging rate increases, the number of POs in directional paging also increases, resulting in substantial gains. The number of POs for 34 bits in PIDP is noticeably less ($2^{6}$) than it was for 32 bits. Thus, for increasing paging rate, the power saving gain for 34 bits shows less variation compared to 32 bits.

In Figure 6b, we show the effect of the increase in the number of beams on the power saving gain (in %) of PIDP for paging rates of 6400 UE/s and UE ID size values of 32,34,36 and 38. As is clear from the analysis, the number of TX beams does not affect either the number of POs or the UE per PO. Therefore, as the number of beams increases, the gain percentage remains constant. Thus, the PIDP mechanism substantiates uniform percentage gains for both a lower, as well as a higher number of beams. Moreover, as expected for a lower value of N2 (32), more gain is manifested compared to the higher value of N2 (38).

## 5. Conclusions

In this article, we propose the Partitioned user ID based Directional Paging (PIDP) mechanism for mmWave-enabled directional 5G communications. The paging resource overheads are significantly higher for directional communications as beam sweeping becomes essential to cover the area of a cell. The proposed PIDP mechanism reduces the UE ID size included in the paging message, which is transmitted over multiple beams, to reduce the paging resource overheads. To ensure that the UE ID information is not compromised in PIDP, the configuration and distribution of UE over various paging occasions are also modified. PIDP manifests around 15% gain with a 20% shorter UE ID when compared to directional paging with the full UE ID included in the paging message transmitted over multiple beams.

## Figures and Tables

**Table 1:** Capacity analysis for directional paging [10].

**Number of Beams**	16	32	64	**LTE**
**System Capacity for Paging (%)**	23.6	47.2	94.4	**1.47**

**Table 2:** Recent related work.

Work Area	Recent Research Works
Paging and tracking area	Mobility model for paging area [11] Rule-based artificial intelligence [13] Multilevel graph partitioning [14] Anchor-based location in HetNets [15] Optimal adjustable extended DRX [16] Interacted cell concept [17]
Paging in MTC and IoT	LTE Group paging for MTC [18] DRX with quick sleeping [19] Reacquisition and synchronization [20] Self-adaptive DRX mechanism [21]
DRX and 5G	Tracking area management for 5G [24] Directional DRX (DDRX) for 5G [23] Hybrid-DDRX [22] Mobile-assisted directional paging [25]

**Table 3:** Parameters for numerical analysis. CRC, Cyclic Redundancy Check; PDCCH, Physical Downlink common Control Channel; RRC, Radio Resource Control.

Parameters	Value
Full UE ID Size (N)	40 bits [26]
Cropped UE Size (N2)	28, 30, 32, 34, 36, 38 bits
Paging Rate, ($R_{pag}$)	1600–6400 UE/s [26]
Number of Beams ($B_{n}$)	16, 32, 64, 128 beams [8]
System Bandwidth, ($\Omega_{w}$)	100 MHz [5]
Cell Edge Spectral Efficiency, ($\eta_{s}$)	0.225 bits/s/Hz [27]
CRC + PDCCH + RRC bits ($\epsilon_{CRC}+\epsilon_{RRC}+b_{PDCCH}$)	64 bits [28]
Time Slot Duration (T)	0.2 ms [29]
